# Weight Fallacies

**Published:** 1914-07

**Authors:** 


### BUFFALO MEDICAL JOURNAL

A Monthly Review of Medicine and Surgery

EDITOR AND PUBLISHER DR. A. L. BENEDICT, 228 Summer St., corner of Elmwood Ave., Buffalo, (Address for all communications. Please make personal and telephone calls before 1 P. M.)

Third, in age, on the western continent. Independent, but supports professional organization. News limited mainly to Western N. Y. and Penn.

Subscription $2.00 a year; $3.00 for two years in advance. Changes and errors in address or failure or duplication of delivery, should be reported immediately.

Yearly Volume 69 JULY, 1914 No. 12

### Weight Fallacies.

The practical importance of the psychic influence of changes in weight, upon both patient and physician, has been suggested by the chagrin of a woman who, in spite of favorable progress, found that she had lost three quarters of a pound in a week. Similar discouragement is often manifested by patients, even those bordering on obesity and, on the other hand, false hopes are often raised by the trivial physiologic variations in diurnal weight. That the medical profession itself is sometimes unduly impressed by changes in weight, is evident from many statements and implications in medical literature and, especially by the almost routine note of gain in weight, in the first reports of every new method of dealing with tuberculosis and by the almost unanimous advice to withhold water in treating obesity, on account of the initial rapid loss of weight thus secured, though it is perfectly obvious that, barring some form of dropsy or other condition actually demanding a restriction of liquids, the metabolic processes on which oxidation of fat depends, require an adequate supply of water.

The late President Lincoln, on being asked how long a man's legs should be, replied that, in his opinion, they should be long enough to reach from the trunk down to the ground. As tables of standard weight and height are almost as freely supplied as blotters, it may be pardonable to state that a man's weight should be somewhere between extreme leanness and the beginning of fatness. Excepting, of course, significant alterations in weight, or rather permanent conditions due to some underlying disease, and limiting our attention to relatively permanent, individual amounts of adipose tissue, there is neither physiologic nor empiric reason for declaring that a certain weight standard should be maintained for a given age or height, although it is convenient to have average figures and a notable departure from any average physical datum, always arouses distrust, even if no definite explanation is at hand.

It is especially important to realize that the popular conception that leanness implies lack of resistance or lack of mental and physical energy is quite incorrect. It has been disproved over and over again in military experience, in studies of longevity, in fact in almost all practical tests of routine endurance. The misconception probably survives because of the confusion of idiosyncratic leanness with emaciation due to disease. It should be stated however, that while most instances of longevity are observed in lean persons, a marked reduction of weight, late in life, in a person previously of fleshy habit, is usually an unfavorable omen, even if no definite pathologic process can be diagnosed.

It must also be conceded that in a great variety of diseases, failures of nutrition of various kinds, reduce the weight below the individual normal. In such conditions, the gradual restoration of the original weight is usually favorable but care must be taken to impress upon the patient, a realization that there is no particular reason for either hoping or desiring that the gain should pass the original norm and that, if this norm has been in excess of the average, it would be better not to pass the latter. Moreover, to avoid discouragement, the attempt should be made to have the patient realize that gain in weight during convalescence should be very gradual and cannot be uninterrupted. in fact, there is as much objection to letting the patient keep track of his own weight as of his own temperatures—on some accounts more.

We have spoken of the diurnal fluctuations of weight as trivial, but it should be clearly understood that they are of greater magnitude than any feasible average gain or loss for a 24-hour period, under comparable conditions. For the sake of convenience, weights are usually taken with the "ordinary" clothing and, for approximate purposes, there is no objection to this plan, provided due allowance is made. Unfortunately, patients—and perhaps physicians—are more apt to be impressed with the importance of a moderate gain or loss from one time to another than with the importance of a fairly exact allowance for clothing. For instance, the man who would be discouraged at a loss of half a pound in a day, would probably pay little attention to whether he wore a hat or a vest at one weighing and not at another, whether he had on low or high shoes, whether he had changed from a winter to a spring suit, whether his pockets were empty or loaded with silver, papers, etc. Exclusive of hat and overcoat, the ordinary winter clothing and the contents of the pockets of a man, may weigh as much as 20 pounds while an outing costume in summer, with empty pockets, may weigh only 5 pounds. It is thus evident that purely extrinsic weights exceed any possible actual gain or loss.

From the theoretic standpoint, we all understand that considerable variations in the alimentary contents may depend upon constipation. While the average weight of the faeces is stated at 60 to 100 grams, it is by no means rare to observe a formed stool a foot long, and over an inch in diameter—30 c. m. long by 3 c. m. in diameter. It is a matter of simple mensuration to show that such a stool has a volume of about 210 c.c. and, according as it floats or sinks in water, its specific gravity corresponds to a weight of a third to half a pound. Constipation involving accumulations of 1 to 3 pounds, is quite an ordinary occurrence.

Equally familiar are the facts that the diurnal urine amounts to 2 to 3 pounds normally, insensible perspiration to 1 to 2 pounds, exhaled watery vapor to nearly as much, that the water drunk in a day amounts to a couple of pounds or more and the solid food to about one pound of proximate organic nutrients and to two or three pounds of gross food stuffs. It requires only a little more elaborate calculation to show that the carbon, exhaled as carbon dioxid, mainly by the breath, amounts to about a third of a pound a day of carbon and to nearly a pound of carbon dioxid.

Unfortunately, the patient, unduly impressed with the idea that a gain or loss of weight means a gain or loss of health, does not understand these factors of diurnal fluctuation; while the physician, knowing them as physiologic data, thinks of them as theoretic and very minor factors, reckoned in grams and miligrams by the physiologic expert, and observed by elaborate instruments. Just to show how large these factors are and how plainly they show themselves without recondite methods of study and by the mere use of ordinary, fairly accurate office scales, the following data are presented:

| | |
|---|---|
| Weight at 11 P. M. with clothing | 167¼ lbs |
| Weight at 11 P. M. naked | 159½ lbs |
| Weight at 11 P. M. naked, after emptying bladder | 159 lbs |
| Weight at 5.30 A. M. naked, before emptying bladder, | 158½ lbs |
| Weight at 5.30 A. M. naked, after emptying bladder, | 157¾ lbs |
| Weight at 9 A. M. naked, before emptying bladder | 157½ lbs |
| Weight at 9 A. M. naked, after emptying bladder | 157 lbs |

In ten hours, without food or drink or exercise or sensible perspiration, we note a total reduction of 2½ pounds in weight, if no allowance is made for the accumulation of half a pound of excreted but not voided urine. Without this allowance, the reduction was two pounds. It will also be noted that more than ¾ of a pound of this reduction was due to evaporation and exhalation, the remainder to urinary loss. Now, while it may seem that in the ordinary weighing of patients during the day time or evening, fairer comparisons may be made, it is obvious that unless attention is given to voidance of urine and faeces, ingestion of food and water, sensible perspiration, not to mention accuracy with regard to clothing, equally great differences may be observed without any change whatever in the actual weight equilibrium.

